# Selected SNPs of *FCN2* Associated with Chronic Tonsillitis in the Polish Adult Population

**DOI:** 10.3390/genes14020242

**Published:** 2023-01-17

**Authors:** Jadwiga Gaździcka, Karolina Gołąbek, Dorota Hudy, Katarzyna Miśkiewicz-Orczyk, Natalia Zięba, Wojciech Tynior, Marek Asman, Maciej Misiołek, Joanna Katarzyna Strzelczyk

**Affiliations:** 1 Department of Medical and Molecular Biology, Faculty of Medical Sciences in Zabrze, Medical University of Silesia, 19 Jordana Str., 41-808 Zabrze, Poland; 2 Department of Otorhinolaryngology and Oncological Laryngology, Faculty of Medical Sciences in Zabrze, Medical University of Silesia, 10 C Skłodowskiej Str., 41-800 Zabrze, Poland

**Keywords:** *FCN2*, single nucleotide polymorphism (SNP), chronic tonsillitis

## Abstract

Chronic tonsillitis is a problem related to bacterial and viral infections. Ficolins play a key role in the defence against various pathogens. In the present study, we investigated the associations between the selected single nucleotide polymorphisms (SNPs) of the *FCN2* gene and chronic tonsillitis in the Polish population. The study included 101 patients with chronic tonsillitis and 101 healthy individuals. The selected SNPs of *FCN2* (rs3124953, rs17514136 and rs3124954) were genotyped using TaqMan SNP Genotyping Assays (Applied Biosystem, Foster City, CA, USA). The analysis of rs17514136 and rs3124953 showed no significant differences in genotype frequencies between the chronic tonsillitis patients and controls (*p* > 0.01). The CT genotype of rs3124954 was significantly more frequent, while the CC genotype was less frequent in chronic tonsillitis patients (*p* = 0.003 and *p* = 0.001, respectively). The frequency of the A/G/T haplotype (rs17514136/rs3124953/rs3124954) was significantly more common in chronic tonsillitis patients (*p* = 0.0011). Moreover, the *FCN2* CT genotype of rs3124954 was associated with a higher risk of chronic tonsillitis, while the CC genotype of rs3124954 decreased this risk. Our findings demonstrate that *FCN2* rs3124954 may be associated with chronic tonsillitis in the Polish adult population.

## 1. Introduction

Palatine tonsils are lymphatic tissues that are essential parts of Waldeyer’s ring, which forms the immune barrier of the respiratory and digestive systems by filtering exogenous antigens and by initiating immune responses [1]. Chronic tonsillitis is diagnosed in adult and children patients and is a global public health problem [2]. Aerobic and anaerobic bacterial and viral infections are connected with tonsillitis [3,4]. Additionally, the microbial biofilm may be crucial in chronic tonsillitis by promoting pathogen survival [1,2]. Patients with chronic tonsillitis may have frequent episodes of sore throats, sleepless nights, fevers and other complaints. Tonsillectomy is still one of the treatment methods that may increase patients’ quality of life [5,6].

The *FCN2* gene encodes L-ficolin (also known as ficolin 2) and is located on chromosome 9p34 [7]. The expression of *FCN2* occurs mainly in the liver. It is also present in the tonsils, bone marrow, small intestine, foetal lung and pancreas but at lower concentrations [8]. Ficolins are part of innate immunity and can recognise pathogen-associated molecular patterns (PAMPs) on pathogens. L-ficolin plays an important role in many viral and bacterial infections. It is also associated with parasites and fungi [9,10]. Additionally, the *FCN2* gene has numerous single nucleotide polymorphisms (SNPs), which are located in promoters, introns or exons [7]. Some of them influence the serum concentrations of L-ficolin (e.g., rs17514136 and rs3124953), while others are associated with the risk of various diseases, such as systemic lupus erythematosus (SLE), schistosomiasis or Chagas disease [11,12,13,14,15,16]. Intronic polymorphisms may have a functional role and may also influence the expression of alternative transcripts of genes or change splicing efficiency [17].

The aim of the study was to evaluate the possible association of selected SNPs (rs17514136, rs3124953 and rs3124954) with chronic tonsillitis in the Polish adult population.

## 2. Materials and Methods

### 2.1. Study Group and Sample Collection

The present study included 101 patients with chronic tonsillitis and 101 healthy individuals. Patients with chronic tonsillitis were recruited at the Department of Otorhinolaryngology and Oncological Laryngology in Zabrze, Medical University of Silesia in Katowice (Poland). All study participants were of Caucasian origin. Data related to age, sex, comorbidities, smoking and drinking were collected using a questionnaire. The control group was sex- and age-matched. All study participants gave their informed consent to be included in the study. The Bioethics Committee of the Medical University of Silesia approved the present study (no. KNW/0022/KB1/49/16 and KNW/0022/KB1/49/II/16/17). The study was conducted in accordance with the Declaration of Helsinki.

Chronic tonsillitis specimens were obtained during tonsillectomy and frozen at −80 °C before DNA isolation. All palatine tonsil samples were verified by a pathologist. Oral epithelial cells were collected from the control group by buccal swabs.

### 2.2. SNPs Selection

The selection of *FCN2* SNPs was based on the minor allele frequency (MAF) in the European population (minimum 0.1) (based on the National Center for Biotechnology Information, dbSNP) [18]. Moreover, all selected *FCN2* SNPs have been analysed in various diseases related to inflammation or infection [11,12,13,14,15,16].

### 2.3. DNA Isolation

Tonsil tissue homogenisation was carried out using the FastPrep^®^-24 instrument (MP Biomedicals, Solon, CA, USA) with Lysing Matrix A tubes (MP Biomedicals, Solon, CA, USA). Genomic DNA was isolated from tonsillar tissue using GeneMatrix Tissue DNA Purification Kits (EURx, Gdańsk, Poland) according to the manufacturer’s protocol. DNA from the swab was extracted using the GeneMATRIX Swab-Extract DNA Purification Kit (EURx, Gdańsk, Poland). A NanoPhotometer^®^ Pearl spectrophotometer (IMPLEN, München, Germany) was used to measure the quality and quantity of extracted DNA.

### 2.4. FCN2 Genotyping

Genotyping of the selected SNPs was performed with a QuantStudio™ 5 Real-Time PCR System and QuantStudio Design and Analysis Software v1.5.1 (Applied Biosystems, Foster City, CA, USA). The primers used in real-time PCR are given in Table 1. The total volume used in the PCR amplification was 25 µL and contained a 11.25 μL DNA sample, a 12.5 μL TaqMan^®^ Genotyping Master Mix (Applied Biosystems, Foster City, CA, USA) and 1.25 μL TaqMan^®^ Genotyping Assays (Applied Biosystems, Foster City, CA, USA). The catalogue numbers for rs17514136, rs3124953 and rs3124954 were C_25765134_10, C_27461651_20 and C_27462209_20, respectively. The PCR conditions were as follows: denaturation at 95 °C for 10 min, 40 cycles of 95 °C for 15 s and 60 °C for 1 min.

### 2.5. Statistical Analysis

STATISTICA v. 13.1 (StatSoft, Krakow, Poland) was used for statistical calculations. The Shapiro–Wilk test was used to check the normality of the age parameter, and the Mann–Whitney U test was used to assess statistical significance between the groups. The SNPs significances were tested by logistic regression. The different-models and haplotype analyses were conducted with SNPStats program [19]. A *p*-value below 0.017 was considered significant; this *p*-value threshold was obtained after Bonferroni correction. All analysed SNPs satisfied the Hardy–Weinberg equilibrium.

## 3. Results

### 3.1. Study Group

The study group of patients with chronic tonsillitis was composed of 101 subjects (56 females and 45 males). The control group included 101 healthy volunteers (56 females and 45 males). Table 2 shows the characteristics of the study group.

Lower age was positively correlated with the risk of chronic tonsillitis (*p* = 0.014). There were no significant difference in smoking status between chronic tonsillitis and controls (*p* > 0.05). Alcohol consumption was negatively correlated with tonsillitis (*p* < 0.01). This may be due to the fact that significantly more individuals in the control group consumed alcohol. We also simultaneously analysed the effect of SNP in men and women on the risk of chronic tonsillitis. However, we found no associations (*p* > 0.05).

Table 3 shows that all analysed SNPs satisfied the Hardy–Weinberg equilibrium.

### 3.2. Distribution of Selected FCN2 SNPs in the Study Group

The *FCN2* genotype distributions are given in Table 4.

The analysis of rs17514136 and rs3124953 showed no significant differences in the frequency of genotypes between chronic tonsillitis patients and controls (Table 4.).

The investigation of rs3124954 showed that the frequency of the CT genotype was significantly higher in chronic tonsillitis patients compared to the controls (*p* = 0.003), while the CC genotype was significantly less prevalent in the study group than in the controls (*p* < 0.001). The TT genotype was at a similar level in the study group and controls.

Our results indicate that the *FCN2* CT genotype of rs3124954 was associated with a higher risk of chronic tonsillitis, while the CC genotype of rs3124954 could decrease the risk of inflammation.

The different-models analysis of the frequency of *FCN2* genotypes in patients with chronic tonsillitis and controls are presented in Table 5.

The haplotypes and their frequencies in the study group are shown in Table 6. The frequency of the haplotype A/G/T (rs17514136/rs3124953/rs3124954) was significantly more common in chronic tonsillitis patients (*p* = 0.0011).

## 4. Discussion

In this study, we investigated the associations between the SNPs of *FCN2* (rs17514136, rs3124953, and rs3124954) and susceptibility to chronic tonsillitis among adult patients in the Polish population.

Some studies found that *FCN2* rs3124953 and rs17514136 were associated with changes in serum L-ficolin [11,20]. Studies found that both *FCN2* SNPs were significantly differently distributed worldwide, depending on ethnicity (e.g., European Caucasians or Brazilians) [21,22]. These ethnic differences in *FCN2* SNPs may influence the concentration and function of L-ficolin and may be associated with different susceptibilities to infections or diseases among different populations [22].

These SNPs were analysed in various diseases, including inflammation among European Caucasians. In this study, we found no significant differences in the genotype frequencies of rs17514136 and rs3124953 between the chronic tonsillitis patients and controls. Similar to our study, Erkan et al. [23] analysed both *FCN2* SNPs in Turkish paediatric patients diagnosed with chronic adenotonsillitis. They found no significant differences in genotype frequencies of these SNPs between patients and controls [23].

Other studies also related to infections found no significant association of *FCN2* rs17514136 and rs3124953 with the risk of dental caries in young Polish patients [24], invasive pneumococcal disease in Caucasians from the United Kingdom [25] or the risk of recurrent respiratory tract infections in Dutch Caucasian children [26]. Additionally, among Japanese patients with inflammatory disorders, no differences were found in the genotype or allele frequencies of rs17514136 and rs3124953 in patients with Behcet’s disease compared to healthy individuals [27]. In addition, these SNPs were not associated with dengue fever or hepatitis B infection in subjects of Vietnamese ethnicity [28,29] or pulmonary tuberculosis in patients from Southeast China [30]. Moreover, none of the genotypes of rs17514136 was associated with susceptibility to rheumatoid arthritis in Brazilian patients [14]. Studies based on the Polish population that analysed the possible associations between rs17514136 and cancer risk showed no association of any genotype of SNP with ovarian cancer [31] or acute myeloid leukaemia [32]. However, the AG genotype and minor G allele in rs17514136 significantly increased the risk of schistosomiasis infection among the Nigerian population, while the AA genotype protected against *Schistosoma haematobium* infection [15]. Elshamaa et al. [33] found that the frequency of the AA genotype of rs17514136 was significantly higher in rheumatic fever (RF) and rheumatic heart disease (RHD) in teenage Caucasian Egyptian patients. Moreover, the A allele was associated with the risk of RF. No association was observed between rs3124953, and the risk of RF or RHD [23]. Marzetti et al. [34] showed that the GG genotype of rs17514136 was a protective factor against RF in Italian children, while the AG genotype protected RF patients against carditis. In addition, the AA genotype of rs3124953 was connected with a late onset of RF [34].

To our knowledge, the present study is the first to investigate the connection between rs3124954 and chronic tonsillitis. Our study showed that the CT genotype of rs3124954 was associated with a higher risk of chronic tonsillitis, while the CC genotype could decrease the risk of tonsillitis. However, Ashmawy et al. [35] investigated rs3124954 in Egyptian children diagnosed with juvenile-onset systemic lupus erythematosus (joSLE). They found that CC homozygotes had a significantly higher risk of joSLE. Moreover, they suggested that the T allele could protect against joSLE [35]. Another study also analysed SLE and rheumatoid arthritis in adult patients in the Brazilian population [14]. No association was observed between the SNPs and these diseases. Instead, the T allele and the TT genotype were significantly associated with nephritis, which is one of the clinical manifestations of SLE [14].

In this study, we found that lower age was associated with a higher risk of chronic tonsillitis. Chronic tonsillitis may be diagnosed at any age. However, it is more common in young adults with the median age of 26–27 years [6,36]. Lower age may be connected to exposure to common pathogens and the developing immune system [4,37].

Moreover, we detected that alcohol was negatively correlated with tonsillitis, which is probably related to the higher number of alcohol consumers in the control group. However, further studies on larger cohorts of patients are warranted to confirm these findings.

Tonsillitis is diagnosed more commonly in women [38,39]. Therefore, we analysed the potential influence of the genotype on the risk of chronic tonsillitis in both sexes. However, we found no significant associations.

The main limitation of the current study was the small sample size. Furthermore, we did not measure L-ficolin serum concentrations, which may have influenced immune defence in patients with chronic tonsillitis. However, we plan to conduct such a study in the future. Moreover, data related to comorbidities were collected using a questionnaire. However, it may not be sufficient to exclude, e.g., asymptomatic liver disease. We should have used a more precise test: ultrasound or assessment of liver enzymes.

## 5. Conclusions

Our findings demonstrate that *FCN2* rs3124954 may contribute to susceptibility to chronic tonsillitis in the Polish population. We suggested that the CT genotype of rs3124954 could increase the risk of chronic tonsillitis. On the contrary, the CC genotype of rs3124954 may decrease this risk. Moreover, the frequency of the A/G/T haplotype was significantly more common in chronic tonsillitis patients. In addition, we reported that chronic tonsillitis was associated with age. Further studies on larger cohorts are warranted to confirm our findings. Furthermore, future investigations are needed to clarify the possible associations between the selected *FCN2* SNPs and clinical manifestations of patients, and L-ficolin serum concentration in patients with chronic tonsillitis.

## Figures and Tables

**Table 1:** Polymorphisms and primers used in genotyping reaction.

SNP ID	Allele Change	SNP Type	Sequence (VIC/FAM)
rs17514136	A/G	intron	GGCACCTTTTGAAGCAAAGACCAGA**[A/G]**GAGATGGAGCTGGACAGAGCTGTGG
rs3124953	A/G	intron	CTCTTCTCTCCTTTCCCTCCTGTTC**[A/G]**TGTGCCCCTGTGCTCTACATACTGC
rs3124954	C/T	intron	GATTCGTGTCAGGATTTCTGGAATG**[C/T]**ATGTGAGACACAGAGCTCTGCGGTG

**Table 2:** Characteristics of the study group.

Characteristic		Chronic Tonsillitis, n (%)	Controls, n (%)
Sex	female	56 (55.45)	56 (55.45)
	male	45 (44.55)	45 (44.55)
Median age (range)		32 (18–64)	37 (18–69)
Smoking	yes	13 (12.87)	21 (20.79)
	no	88 (87.13)	80 (79.21)
Drinking	yes	33 (32.67)	81 (80.20)
	no	68 (67.33)	20 (19.80)
Smoking and drinking	yes	10 (9.90)	21 (20.79)
	no	91 (90.10)	80 (79.21)

**Table 3:** Hardy–Weinberg equilibrium for *FCN2* SNPs.

SNP	Study Group	*p*-Value
rs17514136 (*n* = 198)	All cases	0.368
	Chronic tonsillitis	0.245
	Controls	0.685
rs3124953 (*n* = 199)	All cases	0.816
	Chronic tonsillitis	0.112
	Controls	0.172
rs3124954 (*n* = 194)	All cases	0.999
	Chronic tonsillitis	0.372
	Controls	0.469

**Table 4:** Frequency of *FCN2* genotypes in patients with chronic tonsillitis and controls.

*FCN2* SNP	Variable	Chronic Tonsillitis *n* (%)	Controls *n* (%)	Without Adjustment		Adjusted by Age and Alcohol *	
				OR (95%CI)	*p*-Value	OR (95%CI)	*p*-Value
rs17514136	AA	43 (43.43)	28 (28.28)	0.72 (0.53–0.96)	0.027	0.72 (0.50–1.02)	0.061
	AG	49 (49.50)	52 (52.53)	1.06 (0.80–1.40)	0.700	1.04 (0.75–1.44)	0.830
	GG	7 (7.07)	19 (19.19)	1.77 (1.12–2.79)	0.015	1.83 (1.07–3.11)	0.026
rs3124953	GG	60 (60.61)	70 (70.00)	1.23 (0.92–1.65)	0.165	1.35 (0.96–1.91)	0.085
	AG	38 (38.38)	25 (25.00)	0.73 (0.54–0.99)	0.044	0.66 (0.47–0.95)	0.024
	AA	1 (1.01)	5 (5.00)	2.27 (0.77–6.71)	0.552	2.13 (0.66–6.92)	0.208
rs3124954	CC	41 (41.41)	66 (69.47)	1.79 (1.33–2.41)	1.09 × 10^−4^	1.86 (1.30–2.65)	0.001
	CT	49 (49.50)	25 (26.32)	0.60 (0.45–0.82)	0.001	0.58 (0.40–0.83)	0.003
	TT	9 (9.09)	4 (4.21)	0.66 (0.36–1.22)	0.184	0.62 (0.28–1.35)	0.224

* Adjusted by age and alcohol, becausesmoking and gender do not have significant effects.

**Table 5:** Different-models analysis of frequency of *FCN2* genotypes in patients with chronic tonsillitis and controls.

**rs17514136 Association with Chronic Tonsillitis/Control Probes (*n* = 198, Adjusted by Age and Alcohol)**					
Model	Genotype	Chronic Tonsillitis *n* (%)	Controls *n* (%)	OR (95%CI)	*p*-Value
Codominant	A/A	43 (43.4%)	28 (28.3%)	1	0.028
	A/G	49 (49.5%)	52 (52.5%)	1.61 (0.78–3.34)	
	G/G	7 (7.1%)	19 (19.2%)	4.47 (1.41–14.20)	
Dominant	A/A	43 (43.4%)	28 (28.3%)	1	0.059
	A/G-G/G	56 (56.6%)	71 (71.7%)	1.95 (0.97–3.93)	
Recessive	A/A-A/G	92 (92.9%)	80 (80.8%)	1	0.020
	G/G	7 (7.1%)	19 (19.2%)	3.34 (1.15–9.65)	
Over-dominant	A/A-G/G	50 (50.5%)	47 (47.5%)	1	0.830
	A/G	49 (49.5%)	52 (52.5%)	1.07 (0.56–2.07)	
**rs3124953 association with Chronic tonsillitis/Control probes (*n* = 199, adjusted by age and alcohol)**					
Model	Genotype	Chronic tonsillitis *n* (%)	Controls *n* (%)	OR (95%CI)	*p*-value
Codominant	G/G	60 (60.6%)	70 (70%)	1	0.039
	A/G	38 (38.4%)	25 (25%)	0.46 (0.23–0.95)	
	A/A	1 (1%)	5 (5%)	3.49 (0.32–37.66)	
Dominant	G/G	60 (60.6%)	70 (70%)	1	0.083
	A/G-A/A	39 (39.4%)	30 (30%)	0.55 (0.27–1.09)	
Recessive	G/G-A/G	98 (99%)	95 (95%)	1	0.160
	A/A	1 (1%)	5 (5%)	4.54 (0.43–47.84)	
Over-dominant	G/G-A/A	61 (61.6%)	75 (75%)	1	0.022
	A/G	38 (38.4%)	25 (25%)	0.44 (0.22–0.90)	
**rs3124954 association with Chronic tonsillitis/Control probes (*n* = 194, adjusted by age and alcohol)**					
Model	Genotype	Chronic tonsillitis *n* (%)	Controls *n* (%)	OR (95%CI)	*p*-value
Codominant	C/C	41 (41.4%)	66 (69.5%)	1	0.0022
	C/T	49 (49.5%)	25 (26.3%)	0.30 (0.14–0.63)	
	T/T	9 (9.1%)	4 (4.2%)	0.24 (0.05–1.19)	
Dominant	C/C	41 (41.4%)	66 (69.5%)	1	0.0005
	C/T-T/T	58 (58.6%)	29 (30.5%)	0.29 (0.14–0.59)	
Recessive	C/C-C/T	90 (90.9%)	91 (95.8%)	1	0.220
	T/T	9 (9.1%)	4 (4.2%)	0.38 (0.08–1.81)	
Over-dominant	C/C-T/T	50 (50.5%)	70 (73.7%)	1	0.0026
	C/T	49 (49.5%)	25 (26.3%)	0.34 (0.16–0.69)	

**Table 6:** Haplotype analysis.

**Haplotype Frequencies Estimation (*n* = 202)**							
	rs17514136	rs3124953	rs3124954	Total	Chronic Tonsillitis	Controls	Cumulative Frequency
1	G	G	C	0.3633	0.2982	0.428	0.3633
2	A	G	T	0.2518	0.3244	0.1712	0.6151
3	A	G	C	0.1935	0.1632	0.2258	0.8086
4	A	A	C	0.1689	0.1952	0.149	0.9775
5	G	A	C	0.0176	0.003	0.0259	0.9951
6	G	G	T	0.0049	0.016	0	1
7	G	A	T	0	NA	0	1
8	A	A	T	0	0	0	1
**Haplotype analysis (*n* = 202, adjusted by age and alcohol)**							
	rs17514136	rs3124953	rs3124954	Frequency	OR (95%CI)		*p*-value
1	G	G	C	0.3638	1		---
2	A	G	T	0.2489	0.31 (0.15–0.62)		0.0011
3	A	G	C	0.1949	0.98 (0.47–2.04)		0.950
4	A	A	C	0.17	0.46 (0.23–0.95)		0.038
5	G	A	C	0.0166	1.58 (0.13–19.17)		0.720
rare	*	*	*	0.0058	0.00 (-Inf-Inf)		1.00

Global haplotype association *p*-value: 0.0029. * denotes other haplotypes that occur very rarely or never in our study.

## Data Availability

The data used to support the findings of this research are available upon request.
